# Supplementary material for: Accelerating Payload Release from Complex Coacervates through Mechanical Stimulation
Source: Polymers (Basel). 2023 Jan 23;15(3):586. doi: 10.3390/polym15030586 (PMC9919863; doi:10.3390/polym15030586)
Supplement: Supplementary file 1 [file polymers-15-00586-s001.zip › polymers-2174822 Supplementary Material.pdf]

<u>Supplementary Material</u>

Accelerating Payload Release from Complex Coacervates through Mechanical Stimulation

Wesam A. Hatem and Yakov Lapitsky*

Department of Chemical Engineering, University of Toledo, Toledo, Ohio 43606, USA

*Corresponding Author: yakov.lapitsky@utoledo.edu (ORCID #0000-0003-0063-4233)

A. Continued Swelling of Coacervates After the Release Experiment

When left in deionized water after the release experiments without further agitation, all coacervates (even those subjected to mechanical stimulation) underwent significant further swelling. While a detailed analysis of this post-release experiment effect was beyond the scope of this study, visual observation of samples in the weeks following the experiment revealed all coacervate pellets to markedly increase in volume. This effect is exemplified by the coacervate images in Figure S1 (obtained after the release experiment described in Section 3.1). These images compare the coacervate volumes immediately after the 35-d release experiment (Figure S1a) to those reached after these samples were left in their deionized water release media for another ~ 1 month (without further mechanical stimulation or solvent replacement; see Figure S1b). Both the stimulated and control samples swelled significantly during this period; however, the mechanically stimulated samples (regardless of whether their stimulation occurred through perforation or compression) swelled much less than the control samples.

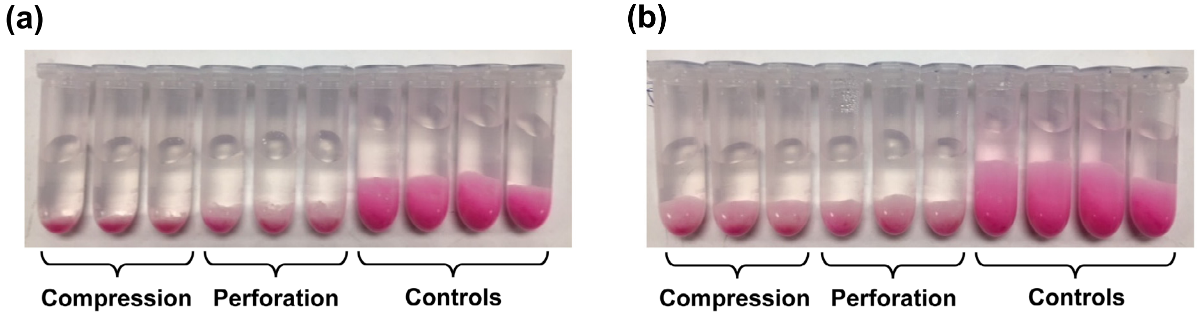

Figure S1. Representative digital photographs of samples (a) at the end of the release experiment, where the samples were stimulated every 3 d, and (b) the same samples after being left in contact with unstirred deionized water (which was not replaced) for approximately one more month. The labels below the images indicate the mechanical stimulation treatments applied to the samples during the release experiment (described in Section 3.1) and the treatment-free controls.
